# Complete genome sequence of *Mycobacterium immunogenum* strain C14-1.MIM isolated from a hospitalized lung transplant recipient

**DOI:** 10.1128/MRA.00618-23

**Published:** 2023-11-09

**Authors:** Fan Jia, Sophie E. Nick, Rebecca M. Davidson, Arthur W. Baker

**Affiliations:** 1 Center for Genes, Environment & Health, National Jewish Health, Denver, Colorado, USA; 2 Duke University School of Medicine, Durham, North Carolina, USA; 3 Division of Infectious Diseases, Duke University School of Medicine, Durham, North Carolina, USA; 4 Duke Center for Antimicrobial Stewardship and Infection Prevention, Durham, North Carolina, USA; University of Maryland School of Medicine, Baltimore, Maryland, USA

**Keywords:** *Mycobacterium immunogenum*, complete genome sequence, lung transplantation

## Abstract

*Mycobacterium immunogenum* is a rapidly growing nontuberculous mycobacteria species that can cause healthcare-associated infections. We report the complete genome of C14-1.MIM, isolated via culture of a respiratory specimen obtained from a hospitalized lung transplant recipient. The circular chromosome contains 5,407,919 bp with a G + C content of 64.24% and 5,217 coding sequences.

## ANNOUNCEMENT

*Mycobacterium immunogenum* is a species of rapidly growing nontuberculous mycobacteria (NTM) that can cause community (1, 2) and healthcare-associated (3–6) infections and outbreaks. Knowledge regarding the genomic content of *M. immunogenum* is limited, and few complete genomes have been reported (7, 8).

We report the complete genome sequence of a new *M. immunogenum* strain, C14-1.MIM. This research was approved by the Duke University Health System Institutional Review Board (Pro00109082), and a waiver of consent was granted.

C14-1.MIM was isolated via mycobacterial culture of a bronchoalveolar lavage (BAL) specimen obtained from a lung transplant recipient. BAL was performed on day 174 of hospitalization in North Carolina, USA. The specimen was decontaminated, concentrated, and inoculated onto a Mycobacteria Growth Indicator Tube, as previously described (9), and mycobacterial growth was noted on day 13. Subculture onto Middlebrook 7H10 media was performed, and *M. immunogenum* identification was performed using matrix-assisted laser desorption ionization-time of flight mass spectrometry.

The whole genome sequence was obtained using both Illumina (Illumina Inc., San Diego, CA) and Oxford Nanopore (Oxford Nanopore Technologies, Oxford, UK) platforms. DNA extraction was performed using the ZymoBIOMICS DNA Miniprep Kit (Zymo Research, Irvine, CA). Illumina libraries were prepared using the NexteraXT Library Prep Kit (Illumina Inc., San Diego, CA), and sequencing was performed using a 2 × 300 bp paired-end configuration on the Illumina MiSeq (MiSeq Control Software version 4.0.0.1769). The run generated 749,084 read pairs with a total of 225,474,284 bases. Raw data were collected using the Illumina Real Time Analysis software 1.18.54.4, with subsequent base calling and demultiplexing using bcl2fastq2 (v2.20). After trimming with Skewer (v0.2.2) (10), the Illumina reads had a mean coverage of 71×.

The Nanopore library was prepared using a Nanopore Native Barcoding Kit 24v12 (SQK-NBD112.24) and sequenced on an Oxford GridION Mk1 (Flow Cell type, FLO-MIN112, R10 Version; Flow Cell ID, FAU07851). DNA was not sheared or size-selected. Primary data acquisition was performed by MinKNOW (v22.05.7) and subsequent base calling by Guppy (v6.1.5), using the high-accuracy algorithm. Adapter trimming was performed with porechop v0.2.3 (11). After trimming, there were 211,419 reads with a total of 706,302,944 bases. The average read length was 3,341 bp (range 2–37,967 bp), and N50 was 4,627 bp. The Nanopore reads had a mean coverage of 131×.

Hybrid assembly with Illumina and Oxford Nanopore reads was performed with Unicycler v0.4.8 (12). All steps, including overlap identification, overlap trimming, and contig rotation were done by Unicycler using default settings. The finished closed genome was rotated to start with dnaA (99.2% identity to gene B1MDH6). The C14-1.MIM genome consists of a 5,407,919 bp circular chromosome with a G + C content of 64.24%. Genome annotation with the National Center for Biotechnology Information Prokaryotic Genome Annotation Pipeline (13) identified 5,217 coding sequences (CDSs), 59 tRNAs, and 6 rRNAs.

Compared to the genomes of the *M. immunogenum* type strain CCUG 47286 (7, 14) and FLAC016 (8), the C14-1.MIM genome is smaller and contains fewer CDSs. Our report, therefore, provides a novel and valuable reference strain to the *M. immunogenum* and NTM research community.

## Data Availability

The complete sequences of the chromosomes of C14-1.MIM have been deposited in GenBank under WGS accession number CP120507 and BioSample SAMN33747153. SRA reads are available under BioProject PRJNA944390. The accession numbers for the SRA reads are SRX19672982 (long reads) and SRX19672981 (short reads).
